# Soluble CD147 (BSG) as a Prognostic Marker in Multiple Myeloma

**DOI:** 10.3390/cimb44010026

**Published:** 2022-01-14

**Authors:** Piotr Łacina, Aleksandra Butrym, Diana Frontkiewicz, Grzegorz Mazur, Katarzyna Bogunia-Kubik

**Affiliations:** 1 Laboratory of Clinical Immunogenetics and Pharmacogenetics, Hirszfeld Institute of Immunology and Experimental Therapy, Polish Academy of Sciences, 53-114 Wrocław, Poland; katarzyna.bogunia-kubik@hirszfeld.pl; 2 Department of Cancer Prevention and Therapy, Wroclaw Medical University, 50-556 Wrocław, Poland; aleksandra.butrym@umw.edu.pl; 3 Department of Haematology, Sokołowski Specialist Hospital, 58-309 Wałbrzych, Poland; d.frontkiewicz@gmail.com; 4 Department of Internal, Occupational Diseases, Hypertension and Clinical Oncology, Wroclaw Medical University, 50-556 Wrocław, Poland; grzegorz.mazur@umw.edu.pl

**Keywords:** BSG, basigin, CD147, MM, multiple myeloma, survival

## Abstract

CD147 (basigin, BSG) is a membrane-bound glycoprotein involved in energy metabolism that plays a role in cancer cell survival. Its soluble form is a promising marker of some diseases, but it is otherwise poorly studied. CD147 is overexpressed in multiple myeloma (MM) and is known to affect MM progression, while its genetic variants are associated with MM survival. In the present study, we aimed to assess serum soluble CD147 (sCD147) expression as a potential marker in MM. We found that sCD147 level was higher in MM patients compared to healthy individuals. It was also higher in patients with more advanced disease (ISS III) compared to both patients with less advanced MM and healthy individuals, while its level was observed to drop after positive response to treatment. Patients with high sCD147 were characterized by worse progression-free survival. sCD147 level did not directly correlate with bone marrow CD147 mRNA expression. In conclusion, this study suggests that serum sCD147 may be a prognostic marker in MM.

## 1. Introduction

Multiple myeloma (MM) is an incurable bone marrow malignancy associated with the presence of atypical plasma cells and with occurrence of end organ damage. It is the second most common haematological malignancy and accounts for 2% of all cancer cases [1,2]. MM is proceeded by monoclonal gammopathy of undetermined significance (MGUS), which has a 1% chance of progressing to malignant MM [3]. While novel therapeutic options, such as autologous stem cell transplantation and immunomodulatory drugs, led in the last two decades to a significant increase in overall survival, mean survival of MM patients is still relatively low at approximately 5 years [4,5].

CD147, also known as basigin (BSG) and extracellular matrix metalloproteinase inducer (EMMPRIN), is a heavily glycosylated member of the Ig superfamily. It is encoded by the *BSG* gene located on chromosome 19p13.3 and is ubiquitously expressed on various types of cells [6,7]. It is primarily described as a transmembrane protein. Its main isoform, basigin-2, is composed of a longer extracellular domain including a signal sequence and two Ig domains, as well as of shorter transmembrane and cytoplasmic domains [8,9]. CD147 can also be found in body fluids in the form of soluble CD147 (sCD147). sCD147 can be secreted by cells as a full-length protein released with microvesicles [10]. Alternatively, the extracellular domain can be cleaved and released through one of two pathways, one involving matrix metalloproteinases (MMPs), and one involving ADAM12 [11,12]. CD147 is a multifunctional protein involved in many cellular pathways [9,13]. Additionally, recent reports indicate that it may function as an alternative entry receptor for the SARS-CoV-2 virus associated with the COVID-19 pandemic [14,15].

CD147 is overexpressed in many cancers and is known to promote cancer progression [16,17]. It was found to be a marker of risk, poor prognosis, overall and progression-free survival, as well as chemotherapy resistance [18,19]. Although many CD147-associated pathways may be responsible for this, it was shown that interaction between CD147 and monocarboxylate transporters (MCTs) contributes most to this pro-tumour effect [20]. MCTs are membrane-bound transporters of monocarboxylates such as lactic acid [21]. They are important components of energy metabolism, as lactic acid is a by-product of glycolysis which needs to be removed from the cell to avoid dangerous decrease in cytosolic pH [22]. Most cancer cells rely primarily on glycolysis for energy production, a phenomenon known as the Warburg effect, making proper functioning of MCTs crucial for them [23]. 

CD147 functions as a chaperone of MCT1 (also known as SLC16A1) and its downregulation is known to be detrimental to proper lactate transport and tumour survival [24,25,26]. CD147 is also known to be involved with expression of other proteins such as MMPs, and vascular endothelial growth factor (VEGF), while its own expression can be controlled by various other factors, e.g., receptor activator for nuclear Factor κ B ligand (RANKL) [27,28,29]. Its ability to induce VEGF expression also contributes to cancer development and progression, as VEGF is an important pro-angiogenic factor [29].

Like in other cancers, CD147 was shown to be overexpressed in MM and to be associated with MM progression [30]. Likewise, MCT1 and MCT4 were also overexpressed in MM patients, although only MCT1 was indispensable for continued MM cell proliferation [24]. Genetic variants of both CD147 and MCT1 were found to influence survival in MM patients [31], and CD147 is known to be involved in response to MM treatment [32,33]. Myeloma cells were observed to exhibit increased lactate transport, whereas CD147 gene expression was found to correlate with key regulators of glycolysis and the Warburg effect, further substantiating the pro-myeloma effect of CD147 [24,34].

Soluble CD147 is thought to support cancer proliferation by interacting with membrane-bound CD147 [35]. It was shown to be an easily detectable biomarker in some diseases [36,37,38], although its role in haematological malignancies is poorly studied. We recently showed thatsCD147 is overexpressed in acute myeloid leukaemia (AML) patients compared to healthy individuals and that high CD147 is associated with worse overall survival [39].

In the present study, we aimed to determine whether serum sCD147 could be used as a potential prognostic marker in MM. Furthermore, we wanted to establish if sCD147 level correlated with the proangiogenic factor VEGF and mRNA expression of CD147 in the bone marrow.

## 2. Materials and Methods

### 2.1. Patients and Controls

The study included 62 newly diagnosed MM patients and 25 healthy blood donors serving as the control group. Both groups were nearly equally divided into men and women (the ratio of females was 30/59 and 12/25, respectively). The study was approved by the Wroclaw Medical University Bioethical Committee (ethical approval code: 369/2019). According to International Staging System (ISS) stratification, 21.4% of patients were in stage I, 33.9% were in stage II, and 44.6% were in stage III. Most patients were administered either the bortezomib, melphalan, prednisone (VMP); 35.2%, or the bortezomib, thalidomide, dexamethasone (VTD); 29.6% regimen as first line therapy. Further clinical data of patients analysed in the study are included in Table 1.

### 2.2. ELISA Analysis of Serum Samples

Peripheral blood from 62 MM patients and 25 healthy individuals was collected, allowed to clot, and subsequently centrifuged for 15 min at 1000× *g*. Serum samples were then collected, aliquoted, and kept at −70 °C until further use. Serum samples were used for measurements of sCD147 and VEGF concentrations, which were performed using the Human EMMPRIN/CD147 Quantikine ELISA Kit and Human VEGF Quantikine ELISA Kit (R&D Systems, Inc., Minneapolis, MN, USA) according to manufacturer’s instructions. All samples were run in duplicate. Subsequently, absorbance was measured in a Sunrise microplate reader with Magellan analysis software (Tecan Trading AG, Männedorf, Switzerland).

### 2.3. RNA Isolation and Gene Expression Analysis

Bone marrow aspirates from a group of 29 MM patients and 3 non-MM patients (working as a PCR control group) were collected, and mononuclear cells were isolated by Lymphodex (inno-train Diagnostik GmbH, Kronberg im Taunus, Germany) density-gradient centrifugation. Total RNA was then extracted using the RNeasy Plus Mini Kit (QIAGEN, Hilden, Germany) according to manufacturer’s protocol. RNA purity and integrity were verified on the DeNovix DS-11 specrophotometer (DeNovix Inc., Wilmington, DE, USA), and by gel electrophoresis. A total of 2000 ng of isolated RNA was used for reverse transcription into cDNA using the High-Capacity cDNA Reverse Transcriptase kit (Applied Biosystems, Waltham, MA, USA) and RNase Inhibitor (Applied Biosystems, Waltham, MA, USA) was added to the reaction mix. The reaction was performed in a SimpliAmp Thermal Cycler (Applied Biosystems, Waltham, MA, USA) according to manufacturer’s instructions. The resulting cDNA was stored at −70 °C.

CD147 (BSG), MCT1 (SLC16A1), MCT4 (SLC16A3), and VEGF gene expression were measured using quantitative real-time PCR, and the raw expression data were normalized to β-actin (ACTB), which was used as a reference gene. TaqMan Gene Expression assays specific to each gene of interest, as well as TaqMan Gene Expression Master Mix (Applied Biosystems, Waltham, MA, USA) were used for the experiment. TaqMan Gene Expression probes (Applied Biosystems, Waltham, MA, USA) used were: Hs00936295_m1 (CD147), Hs01560299_m1 (MCT1), Hs00358829_m1 (MCT4), Hs00900055_m1 (VEGF), and Hs01060665_g1 (ACTB). Samples were run in duplicate. The reactions were performed on LightCycler 480 II (Roche Diagnostics, Rotkreuz, Switzerland) and according to manufacturer’s instructions. Relative expression was then calculated using the 2^−ΔΔCt^ method.

### 2.4. Statistical Analysis

The non-parametric Mann–Whitney U test was used for comparison between serum sCD147 and clinical parameters such as white blood cell count, haemoglobin, total protein, albumin, lactate dehydrogenase, β2-microglobulin, creatinine or C-reactive protein. Spearman’s coefficient was used to assess correlations with serum sCD147, serum VEGF, CD147/MCT1/MCT4/VEGF gene expression, and clinical parameters. Overall and progression-free survival were analysed using the Kaplan–Meier curves and Gehan–Breslow–Wilcoxon test, as well as multivariate Cox proportional hazards model, and the non-parametric Wilcoxon signed-rank test was employed to compare sCD147 level before and after response to treatment. These analyses were performed with the Real Statistics Resource Pack for Microsoft Excel 2013 (version 15.0.5023.1000, Microsoft, Redmond, WA, USA), RStudio (RStudio, PBC, Boston, MA, USA), and GraphPad Prism (version 8.0.1, GraphPad Software, San Diego, CA, USA). *p*-values < 0.05 were considered statistically significant, while those between 0.05 and 0.10 were indicative of a trend.

## 3. Results

### 3.1. Serum Soluble CD147 Is Increased in MM Patients

**Figure 1:**
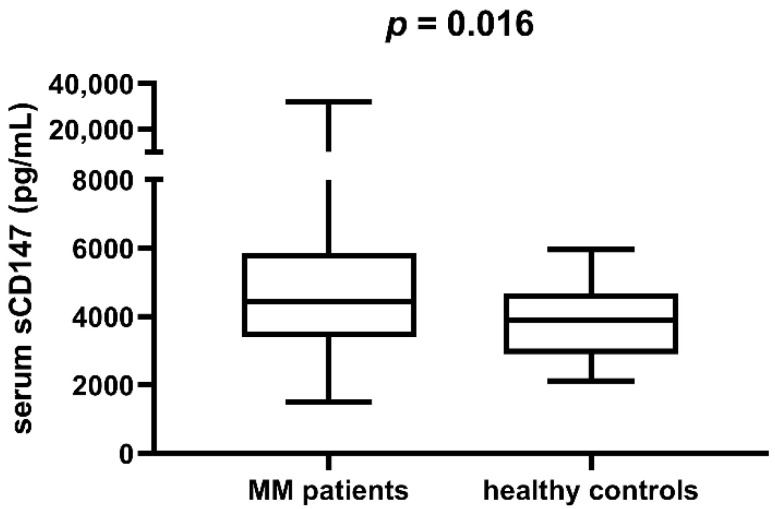
Serum sCD147 in multiple myeloma (MM) patients and the control group of healthy individuals. sCD147 is higher in the former group (*p* = 0.016).

We analysed expression of sCD147 in serum of a group of MM patients (*n* = 62) and in the control group of healthy individuals (*n* = 25). The median sCD147 value was 4441.78 pg/mL (interquartile range: 3435.10–5798.96 pg/mL) in patients, and 3894.45 pg/mL (interquartile range: 2903.50–4544.15 pg/mL) in the control group. We found sCD147 to be significantly higher in serum of MM patients compared to the control group (*p* = 0.016, Figure 1).

### 3.2. Serum Soluble CD147 Is Associated with More Advanced Disease and Worse Survival

**Figure 2:**
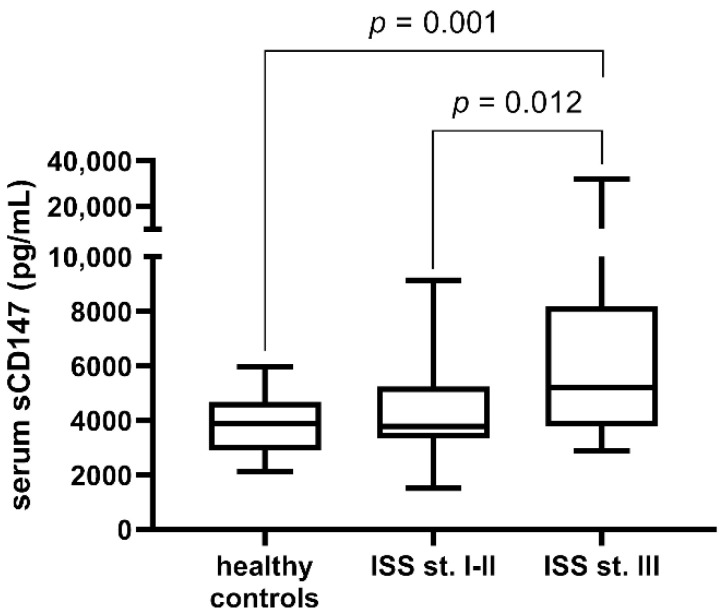
Serum sCD147 in patients in stages I-II and in patients in stage III of the International Staging System, or ISS. Healthy controls are also included. Patients in the more advanced stage III are characterized by higher sCD147 levels than both patients in the stages I-II (*p* = 0.014), and healthy controls (*p* = 0.002). Concurrently, healthy individuals did not significantly differ from patients in stages I-II (*p* = 0.279).

We analysed sCD147 expression in the context of some of the clinical parameters of MM. sCD147 was higher in patients in the more advanced stage III (mean: 5202.93 pg/mL, interquartile range: 3909.71–7986.53 pg/mL), compared to patients in stages I-II (mean: 3782.70, interquartile range: 3352.70–5196.10 pg/mL), according to the International Staging System (ISS) criteria (*p* = 0.012, Figure 2). sCD147 expression in stage III patients was also significantly higher than that of healthy individuals (*p* = 0.001).

**Figure 3:**
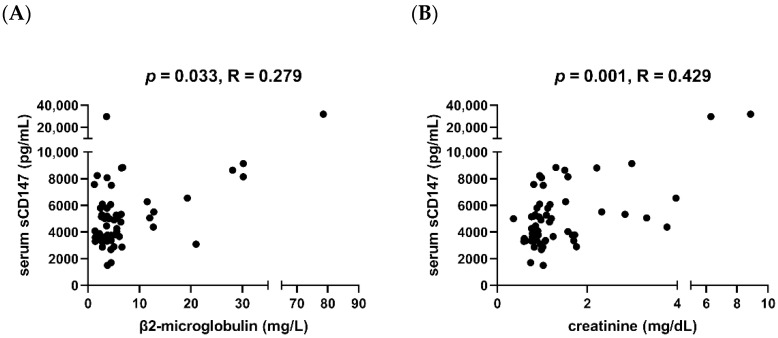
Correlation between serum soluble CD147 and two clinical parameters in MM patients—β2-microglobulin (**A**) and creatinine (**B**). Serum soluble CD147 is characterized by a weak-moderate correlation with these two parameters.

Regarding other clinical parameters, we observed that sCD147 correlated with β2-microglobulin level (R = 0.279, *p* = 0.033, Figure 3A) and creatinine (R = 0.429, *p* = 0.001, Figure 3B). However, no associations with either white blood cell count, haemoglobin, total protein, albumin, lactate dehydrogenase, or C-reactive protein were observed. Additionally, we compared serum levels of sCD147 and of the proangiogenic factor VEGF. However, we did not find any correlation between them (R = −0.010, *p* = 0.941).

**Figure 4:**
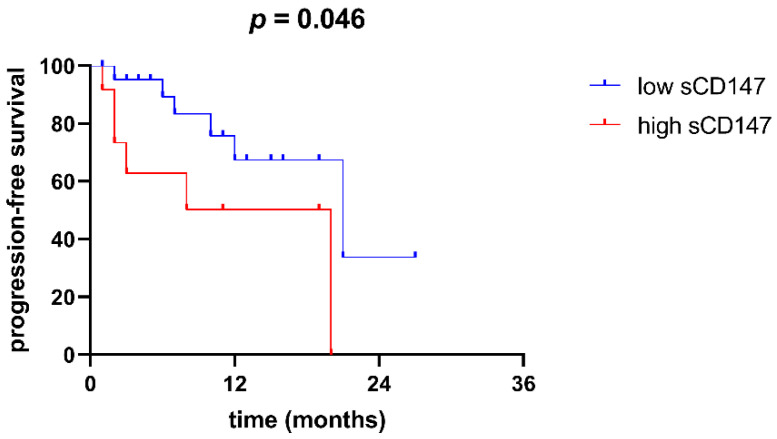
Progression-free survival of patients with high serum sCD147 (above the upper quartile, or 5798.96 pg/mL) and in patients with low serum sCD147 (below the upper quartile). Patients with high sCD147 are characterized by a more adverse PFS (*p* = 0.046).

In the next step, we used Kaplan–Meier curves to analyse the difference in overall (OS) and progression-free survival (PFS) between patients with high and low sCD147. High sCD147 was defined in this and further analyses as being above the upper quartile in our study group (5798.96 pg/mL), and low sCD147 was below the upper quartile. While no difference was observed in OS, we observed that patients with high sCD147 were characterized by shorter PFS than patients with low sCD147 (*p* = 0.046, Figure 4). 

Additionally, we constructed a Cox proportional hazards model including sCD147 status (low/high) and adjusting for age, β2-microglobulin level, creatinine level, ISS stage, and therapy (use of immunomodulatory drugs). This analysis confirmed high sCD147 to be an independent marker of adverse PFS (*p* = 0.038).

### 3.3. Serum Soluble CD147 Levels Drop in Response to Treatment

**Figure 5:**
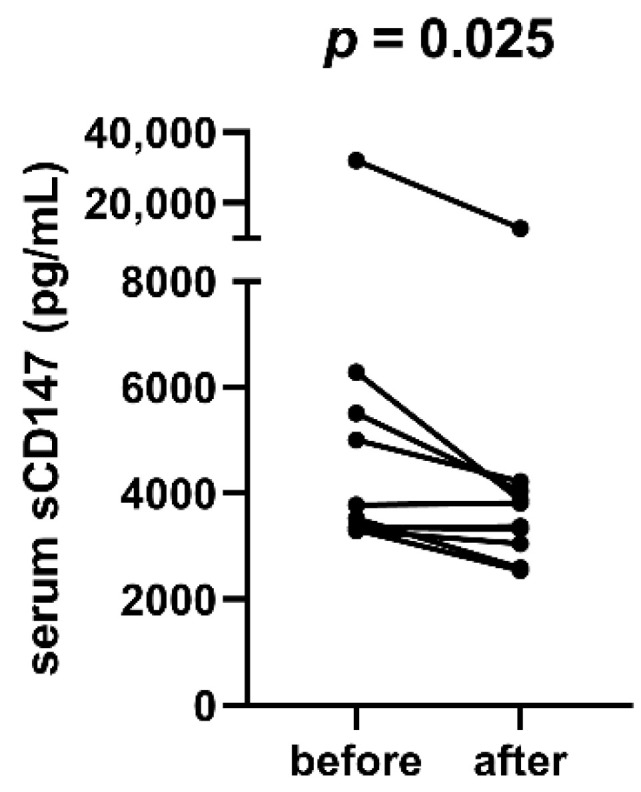
Serum sCD147 levels in multiple myeloma patients before and after positive response to treatment. sCD147 levels are decreased in most patients after remission (*p* = 0.025).

In a subsection of MM patients (*n* = 10), we compared sCD147 levels after positive response to treatment (very good partial response or better) to those at diagnosis. We found sCD147 levels to significantly decrease in response to treatment in most patients (*p* = 0.025, Figure 5).

### 3.4. Serum Soluble CD147 Does Not Correlate with BSG mRNA Expression Levels

We measured relative mRNA *BSG* expression in bone marrow samples of a subgroup (*n* = 29) of MM patients. sCD147 level in serum did not correlate with mRNA CD147 expression in those patients (R = 0.027, *p* = 0.896). In addition to BSG, we also measured mRNA expression of various other genes associated with BSG in MM. We found that, as expected, BSG mRNA expression correlated strongly with expression of VEGF (R = 0.420, *p* = 0.023), MCT1/SLC16A1 (R = 0.811, *p* < 0.001), but not with MCT4/SLC16A3 (R = 0.061, *p* = 0.755). While no statistically significant associations between mRNA BSG expression and clinical parameters or survival were observed, we found a trend towards lower BSG expression in patients with positive (very good, partial, or better) response to treatment (*p* = 0.093).

## 4. Discussion

A growing body of evidence points to CD147 being a major factor in cancer progression and survival [16,17,18,19]. This may be a result of its multifunctional nature and involvement in a multitude of regulatory pathways that include cell migration, proliferation, and angiogenesis. Another pathway associated with tumour progression and promoted by CD147 is epithelial–mesenchymal transition (EMT) [40,41]. Furthermore, recent research suggests that control of lactate transport through MCTs is a major function of CD147 in the context of cancer [20]. As CD147 was recently shown to function as an auxiliary receptor for SARS-CoV-2 infection [14,15], a good knowledge of its expression patterns may be of importance in the context of the COVID-19 pandemic. 

Various studies suggests that CD147 is involved in development, progression, and response to treatment of MM [24,30,42]. CD147 is also implicated in response to treatment, particularly treatment involving immunomodulatory drugs [32,33]. In our previous studies, we showed that some genetic variants of CD147 and MCT1 affect survival of MM patients [31]. Soluble CD147 is a form of CD147 found in all body fluids and an easily measured potential biomarker, which we recently showed to be associated with survival in AML patients [39]. However, little is known about sCD147 in MM patients.

The main goal of the present study was to establish if sCD147 could be a prognostic marker in MM. Earlier study showed that serum/plasma sCD147 is elevated in many diseases, including some cancers [36,37,38,43,44] and our earlier studies show that it is elevated in AML [39]. Here, we observed that sCD147 level is significantly higher in MM patients compared to healthy individuals. Furthermore, sCD147 is higher in patients with more advanced disease than in both patients with less advanced MM, and healthy individuals. Additionally, we showed that sCD147 dropped in most analysed patients as a result of achieving remission, regardless of treatment regimen. High sCD147 also predicted shorter PFS, independently of variables such as age, ISS stage, or treatment regimen (whether immunomodulatory drugs were used or not). 

All this evidence suggests that sCD147 is a potential prognostic marker associated with MM in general, and more particularly with MM progression and survival. Our results resemble those found in a study on breast cancer, which showed higher sCD147 in patients with primary breast cancer compared to benign diseases, as well as in patients with advanced cancer compared to early stage disease [37]. Similarly, sCD147 was shown to be elevated in patients with hepatocellular carcinoma (HCC), and to correlate with HCC tumour size and worse survival [36,45]. 

In our previous study, we likewise demonstrated that sCD147 is higher in AML patients and correlates with various AML clinical parameters and survival [39]. However, data on sCD147 expression in other cancers are scarce. Given that sCD147 is abundant in serum and plasma, but also in body fluids such as saliva and urine, it can be easily measured by protein detection methods such as ELISA. Therefore, it appears to be an interesting candidate for a potential biomarker not just in MM, but in cancer in general.

The role of sCD147 in multiple myeloma, and in cancer in general, is not very clear. CD147-containing microvesicles were shown to be internalized by myeloma cells and to increase their proliferation [42]. sCD147 is known to dimerize with membrane-bound CD147 in the tumour microenvironment, to stimulate production of more CD147 as well as various other proteins enhancing tumour survival [35,46]. However, we did not observe sCD147 to correlate with levels of the pro-angiogenic factor VEGF, even though there was a correlation between VEGF and CD147 expression on the mRNA level. This suggests that sCD147 might not be involved in BSG-dependent induction of VEGF expression and angiogenesis [29]. However, it is worth noting that VEGF regulation is very complex and involves many factors and pathways [47]. This includes post-transcriptional regulation processes, which means that its mRNA expression may not necessarily correspond to actual protein expression [48]. Additionally, we did not find the sCD147 level to correlate with CD147 mRNA expression. However, mRNA expression of both VEGF and MCT1 (SLC16A1) did correlate with CD147 mRNA level, as expected [29,33]. This may be due to the fact that CD147 is primarily expressed on the cell surface. Only a part of it is secreted, and this secretion is dependent on three specialised secretion pathways (one involving microvesicles, and two involving shedding of the CD147 extracellular domain) [10,11,12]. These results suggest that sCD147 secretion may not be the main way for CD147 to exert its pro-tumour effect, although sCD147 still appears to be an interesting biomarker in multiple myeloma.

In conclusion, we showed that sCD147 may be a prognostic marker in MM, although our results may require confirmation on a larger cohort of patients. sCD147 appears to be a promising marker of cancer.

## Figures and Tables

**Table 1:** Characteristics of MM patients included in the study.

Data	Median and Range (*n* = 59)
Age	70 (43–88)
white blood cell count (G/L)	6.7 (2.4–20.9)
haemoglobin (g/dL)	10.1 (5.6–14.7)
total protein (g/dL)	8.4 (5.1–15.9)
albumin (g/dL)	3.8 (1.7–5.0)
lactate dehydrogenase (U/L)	225 (100–1595)
β2-microglobulin (mg/L)	4.4 (1.3–78.5)
C-reactive protein (mg/L)	10.2 (0.7–102.8)
creatinine (mg/dL)	1.00 (0.36–8.87)
calcium (mg/dL)	9.4 (7.4–14.0)

## Data Availability

The data presented in this study are available on request from the corresponding author. The data are not publicly available due to privacy or ethical restrictions.
